# Early-Life Stress Does Not Aggravate Spatial Memory or the Process of Hippocampal Neurogenesis in Adult and Middle-Aged APP/PS1 Mice

**DOI:** 10.3389/fnagi.2018.00061

**Published:** 2018-03-07

**Authors:** Lianne Hoeijmakers, Anna Amelianchik, Fleur Verhaag, Janssen Kotah, Paul J. Lucassen, A. Korosi

**Affiliations:** Brain Plasticity Group, Center for Neuroscience, Swammerdam Institute for Life Sciences, University of Amsterdam, Amsterdam, Netherlands

**Keywords:** early-life adversity, neuroplasticity, Alzheimer’s disease, neurogenesis, Morris water maze, hippocampus, cognition

## Abstract

Life-time experiences are thought to influence the risk to develop the neurodegenerative disorder Alzheimer’s disease (AD). In particular, early-life stress (ES) may modulate the onset and progression of AD. There is recent evidence by our group and others that AD-related neuropathological progression and the associated neuroimmune responses are modulated by ES in the classic APPswe/PS1dE9 mouse model for AD. We here extend our previous study on ES mediated modulation of neuropathology and neuroinflammation and address in the same cohort of mice whether ES accelerates and/or aggravates AD-induced cognitive decline and alterations in the process of adult hippocampal neurogenesis (AHN), a form of brain plasticity. Chronic ES was induced by limiting bedding and nesting material during the first postnatal week and is known to induce cognitive deficits by 4 months in wild type (WT) mice. The onset of cognitive decline in APP/PS1 mice generally starts around 6 months of age. We here tested mice at ages 2–4 months to study acceleration and at ages 8–10 months for aggravation of the APP/PS1 phenotype. ES-exposed WT and APP/PS1 mice were able to perform the object recognition (ORT) and location tasks (OLT) at 2 months of age. Interestingly, at 3 months, ES induced impairments in the performance of the OLT in WT, but not in APP/PS1 mice. APP/PS1 mice exhibited alterations in hippocampal cell proliferation and differentiation, but ES exposure did not further change this. At 9 months, APP/PS1 mice exhibited impaired performance in the Morris Water Maze (MWM) task, as well as reductions in markers of the AHN process, which were not further modulated by ES exposure. In addition, we observed a so far unreported hyperactivity in ES-exposed mice at 8 months of age, which hampered assessment of cognitive functions in the ORT and OLT. In conclusion, while ES has been reported to modulate AD neuropathology and neuroinflammation before, it failed to accelerate or aggravate the decline in cognition or the process of AHN in APP/PS1 mice at ages 2–4 and 8–10 months. Future studies are needed to unravel how ES might affect the vulnerability to develop AD.

## Introduction

Alzheimer’s disease (AD) is the most prevalent form of dementia in elderly. It is characterized by an age-related accumulation of amyloid β (Aβ) neuropathology and tau-related neurofibrillary tangles in the brain (Querfurth and LaFerla, 2010; Scheltens et al., 2016). While mutations in the amyloid or tau genes induce familial AD in a few percent of the patients, gene-environment interactions likely play an important role in the majority of patients with sporadic AD (Mayeux and Stern, 2012). Indeed, multiple life-style factors have been described to modulate AD age-of-onset and progression (Okonkwo et al., 2014; Külzow et al., 2016). For example, stress experiences in elderly were a potent accelerator of age-related cognitive decline (Aggarwal et al., 2014) and the total amount of life-time distress was in addition associated with an aggravated age-related cognitive decline and AD development (Wilson et al., 2003, 2006, 2007; Johansson et al., 2014; Sindi et al., 2017).

Recent clinical studies have further suggested that exposure to stress or trauma early in life might enhance the vulnerability to develop dementia later in life (Seifan et al., 2015; Wang et al., 2016), although the study of this hypothesis has only recently begun. The early-life period is a sensitive time for brain development, during which the brain is particularly vulnerable to adversities, that can alter developmental trajectories and lead to life-long changes in brain function (Heim and Nemeroff, 2001; Barker, 2004; Barker et al., 2013). In fact, early-life stress (ES) has been shown to induce deficits in adult hippocampal structure and functioning, as well as cognitive impairments in both humans and rodent ES models (Staff et al., 2012; Naninck et al., 2015; Wang et al., 2016; Calem et al., 2017).

Interestingly, accumulating preclinical evidence indicates that ES might also modulate the progression of Aβ pathology. For example, processing of the amyloid precursor protein (APP) was increased in non-transgenic adult rats exposed to maternal separation stress (Solas et al., 2010, 2013; Martisova et al., 2012, 2013). In addition, ES aggravated Aβ pathology in 4-month-old bi-genic APP and Tau mutant (biAT) mice and in APPswe/PS1dE9 mice at 9–10 months of age, two models that both overexpress AD related genetic variants and present with AD related neuropathology (Lesuis et al., 2016; Hoeijmakers et al., 2017; Hui et al., 2017). These ES-induced alterations in Aβ pathology have also been associated with altered Aβ-induced neuroinflammatory signaling and microglial activation in APP/PS1 mice (Hoeijmakers et al., 2017).

There is, furthermore, initial evidence that such alterations in AD-related neuropathology are also associated with a stronger impairment in spatial memory in 9-month-old, maternally stressed APP/PS1 mice, when compared to unstressed APP/PS1 mice (Hui et al., 2017). However, relative to the unstressed transgenic mice, 4-month-old APP/PS1 mice exposed to prenatal stress, or chronic ES-exposed 4-month-old biAT mice, showed reduced cognitive deficits or no differences in cognition, respectively (Sierksma et al., 2013; Lesuis et al., 2016). This indicates that the consequences of ES for cognition might depend on the specific model or ages studied.

One structural substrate implicated in hippocampal plasticity and cognition is adult hippocampal neurogenesis (AHN), which refers of the generation of new neurons in the hippocampus, that can be studied by the expression of various stage-specific cellular markers. Various APP-based mouse models have demonstrated reductions in the numbers of neurogenic cells in their hippocampus (Donovan et al., 2006; Rodríguez et al., 2008; Demars et al., 2010). As neurogenesis could respond to Aβ neuropathology as well as the related neuroinflammation (Biscaro et al., 2012; Varnum et al., 2015; De Lucia et al., 2016), one could hypothesize that an ES-induced aggravation of Aβ and the altered neuroinflammatory profile might also further affect the process of AHN. Together, such ES-induced changes in the hippocampus might contribute to an acceleration or aggravation of cognitive decline.

We therefore here set out to study whether ES-induced changes in AD hallmarks, that we and others have characterized before, are also associated with accelerated and/or aggravated cognitive deficits and impaired neuronal plasticity. To this end, we exposed the well-characterized APPswe/PS1dE9 mouse model to chronic ES from postnatal day (P)2 to P9 to test if ES-exposed AD mice exhibit an earlier onset of cognitive decline (i.e., already at 2–3 months of age), or whether ES exposure might aggravate cognitive deficits at later ages. We further studied if ES might affect the APP/PS1-related alterations in the process of AHN. Finally, we tested if these measures correlate with one another as well as with the previously reported changes in AD-related hallmarks (including Aβ pathology and microglial markers Iba1 and CD68) in this cohort (Hoeijmakers et al., 2017).

## Materials and Methods

### Mice and Early-Life Stress Paradigm

Bigenic APPswe/PS1dE9 hemizygous male mice on a C57BL/6J background and their wild type (WT) littermates were used in this study, as described previously (Hoeijmakers et al., 2017). All animals were bred in house and underwent the chronic ES paradigm, consisting of limiting the nesting and bedding material for 1 week (Naninck et al., 2015; Hoeijmakers et al., 2017).

Briefly, the dams and pups were assigned to the ES or control (Ctrl) condition on P2. They were left undisturbed until P9 and moved to standard housing cages until weaning at P21. The standard housing consisted of cage enrichment, ad libitum water, standard chow, 20–22°C temperature, and 40%–60% humidity. All mice were housed with 2–4 same-sex littermates per cage. Experimental procedures were conducted according to the Dutch national law and European Union directives on animal experiments and were approved by the animal welfare committee of the University of Amsterdam.

### Experimental Design

The mice were tested for cognitive functioning in various behavioral tests. Behavior was investigated in a first cohort of mice (cohort 1) to test if ES impairments were accelerated in APP/PS1 mice and therefore already present at 2 months of age. A second cohort of mice (cohort 2) was tested for accelerated decline in one behavioral task at 3 months, and sacrificed at 4 months for further analyses. A third cohort of mice (cohort 3) was used for behavioral testing at 8 months of age. Finally, cohorts 1 and 3 were used to investigate if ES aggravated cognitive impairments at 9 months of age, and they were sacrificed at 10 months of age for further analyses. All mice were injected with the cell-birth date marker 5-bromo-20-deoxyuridine (BrdU) prior to sacrifice (see “Tissue Collection and Processing” section).

Cohort 1 included 45 mice (Ctrl WT *n* = 9 of 7 litters, ES WT *n* = 12 of 8 litter, Ctrl APP/PS1 *n* = 13 of 7 litters, ES APP/PS1 *n* = 16 of 7 litters). Cohort 2 included 38 mice (Ctrl WT *n* = 10 of 5 litters, ES WT *n* = 11 of 5 litter, Ctrl APP/PS1 *n* = 9 of 5 litters, ES APP/PS1 *n* = 8 of 4 litters). Cohort 3 included 20 mice (Ctrl WT *n* = 6 of 3 litters, ES WT *n* = 8 of 5 litter, Ctrl APP/PS1 *n* = 4 of 3 litters, ES APP/PS1 *n* = 4 of 4 litters). A total of 36 mice from cohort 1 underwent additional behavioral testing at 9 months (Ctrl WT *n* = 9 of 7 litters, ES WT *n* = 12 of 8 litter, Ctrl APP/PS1 *n* = 8 of 5 litters, ES APP/PS1 *n* = 7 of 5 litters).

### Behavior

Behavioral testing was performed as previously described (Naninck et al., 2015). The object recognition task (ORT) is a non-spatial, emotionally neutral memory test that makes use of the inherent curiosity and novelty-seeking behavior of mice. The object location task (OLT) is a spatial, emotionally neutral memory test, which like the ORT, depends on novelty-seeking behavior. The elevated plus maze (EPM) is used to address basal exploration and anxiety-like behavior. The Morris Water Maze (MWM) is a spatial memory task in which mice can escape the water bath by locating a hidden platform using spatial cues.

The behavioral tasks were performed during the dark (lights-off) phase to accommodate to the natural, active period of mice, and they were therefore moved at least 1 month prior to testing, to a housing room with a reversed 12/12 day/night cycle (8 AM lights off). The testing room was lid by three red-light spots (25 W), and mice were transferred daily to the room 1 h prior to testing. Prior to behavioral testing, mice were handled for 2 days in the housing room and for 2 days in the testing room.

The behavioral performance of the mice was recorded and tracked for automated analysis of locomotion and position using Ethovision software (Noldus, Wageningen, Netherlands). ORT and OLT object exploration behavior and EPM arm exploration behavior were scored using Observer software (Noldus, Wageningen, Netherlands), by an investigator who was unaware of the experimental conditions.

#### ORT

The testing-box for the ORT was a rectangular blue-plastic box (l × w × h: 23.5 × 33.1 × 27 cm) with a sawdust covered bottom, cleaned in between trials with 25% ethanol. The ORT consisted of four consecutive days with on each day a 5-min trial; two habituation trials during which the mice freely explored the testing-box, one training trial when the mice explored two identical objects in the testing-box, and one testing trial 24-h after the training phase. One of the objects was replaced during this testing trial, and the mice were allowed to explore this novel object and the old, familiar object during the 5-min trial.

Locomotion in the box was tested on all days as an indicator of basal exploration behavior. Mice that spent <10 s exploring the objects during the training or testing trial were excluded from the analysis. The exploration time of the objects during the training phase and the testing phase was scored to test if this ratio was equal to 1, indicating no prior preference or bias. The novel object exploration time over familiar object exploration time was calculated after the testing phase. A ratio for novel/familiar exploration time that was >1 indicated a preference for the novel object and discrimination from the familiar object.

#### OLT

The OLT task was performed in the same box as the ORT, and likewise consisted of 5-min trials on 4 days with sequentially two habituation trials, one training trial and 24-h later the testing trial. During the training trial, the mice were exposed to two new identical objects placed in the middle of the box, of which one was replaced to a new location during the testing trial. If mice recognized this novelty on the testing day, they were expected to explore the object in the novel location more so than the familiar location.

Locomotion in the box was tested on all days as an indicator of basal exploration behavior. Mice that spent <10 s exploring the objects at the different locations during the training or testing trial were excluded from the analysis. Similar to the ORT, the ratio of object exploration time during the training trials should be equal to 1, and a preference for the novel location was indicated by the novel/familiar location exploration time >1.

#### EPM

The EPM was a plus-shaped maze with a neutral 5 × 5 cm center and 35 × 5 cm long arms, raised 100 cm above the floor. Two opposed arms were enclosed with a 30-cm wall, and the two other arms without any enclosure were open arms (OAs). Every mouse was placed in the center facing one of the OA and allowed to freely explore the maze for 10 min. One animal fell of the maze and was therefore excluded from further analysis. Locomotion during the EPM was tested on all days as an indicator of anxiety and basal exploration behavior. OA exploration time and entries were calculated as a percentage of total arm time or entries.

#### MWM

We addressed learning behavior during acquisition trials in the MWM and flexibility of this learning behavior during reversal training. We additionally adapted the MWM protocol to make the reversal training more difficult with the use of a smaller platform during reversal training, which requires more precise spatial navigation of the mice (Vorhees and Williams, 2006).

The MWM was a circular pool (110 cm diameter) filled with water (24 ± 1°C). For acquisition and reversal training, the mice underwent twice daily, 1-min trials with a 30-min inter-trial time by placing them at different, random starting positions in this pool, to exclude egocentric learning strategies. After a trial, the mice were placed in a clean cage in front of an infrared (heating) lamp for ±1 min to prevent hypothermia.

The protocol started with 1 day of cued trials, during which the pool was filled with clear water and a visible 12-cm diameter platform was placed in the center of the pool. When the mice did not locate the platform within the 1-min trial, they were guided to and placed on the platform for 15 s. During the following 6 days of acquisition trials, the pool was surrounded by spatial cues to support allocentric spatial navigation, the water was adjusted to opaque by addition of non-toxic paint and the platform was submerged just below the water-surface in a fixed position within target quadrant 1 (Tq1). For the probe trial on day 8, the platform was removed and the mice were placed in the pool for the full 1-min trial. The protocol then continued with 5 days of reversal trials with a platform that was reduced to half the original size (6-cm diameter) and placed in a new location in the maze within Tq2. A second memory trial followed reversal training on day 14.

The latency to reach the platform (escape latency) was measured manually during acquisition and reversal training. Three mice floated throughout the acquisition and/or reversal trials and were excluded from the analysis. The time spent in Tq1 and Tq2 was recorded during the respective probe trials on day 8 and day 14, as well as locomotion behavior during all trials.

### Tissue Collection and Processing

To ensure that brain plasticity measurements reflected basal levels, the mice of cohorts 2 and 3, and several mice from cohort 1 (2 Ctrl WT, 1 ES WT and 2 Ctrl APP/PS1 mice) were sacrificed 4 weeks after behavioral testing, at either 4 months (cohort 2) or 10 months of age (cohort 1 and 3). The mice of cohort 1 were included to balance the group numbers for the analysis of brain tissue at 10 months of age and were randomly selected. The mice were injected intraperitoneally prior to sacrifice with the cell birth date marker 5-bromo-20-deoxyuridine (BrdU, Sigma-Aldrich), dissolved in 0.9% saline containing 0.007 M NaOH. Four-month-old mice received three pulses of 100 mg/kg BrdU with a 2-h interval on two consecutive days. Ten-month-old mice received three pulses of 100 mg/kg BrdU with a 2-h interval on three consecutive days. Two hours after the last BrdU injection, the mice were sacrificed by transcardial perfusion with paraformaldehyde for later immunohistochemical purposes as previously described (Naninck et al., 2015; Hoeijmakers et al., 2017). Tissue was processed to obtain 40 μm coronal sections in six parallel series as described before Naninck et al. (2015) and Hoeijmakers et al. (2017).

### Immunolabeling for Adult Hippocampal Neurogenesis Markers

#### Antibodies

BrdU immunofluorescent labeling was obtained with a 1:500 dilution of the rat anti-BrdU antibody (Accurate Chemical and Scientific Corporation OBT0030, Westbury, NY, USA) and doublecortin (DCX) immunohistochemical labeling was obtained with a 1:800 dilution of the goat anti-DCX antibody (SantaCruz Biotechnology, Dallas, TX, USA). Immunolabeling for both antibodies followed previous descriptions (Naninck et al., 2015; Hoeijmakers et al., 2018).

#### Quantification

An even representation of the hippocampus over the rostral-caudal axis was obtained by selecting 6 coronal, bilateral sections of the hippocampus with 300 μm intersection distance, therewith including three sections rostral of Bregma −2.30 mm and three sections caudal of Bregma −2.30 mm. All quantifications were performed by a researcher blind to the experimental conditions.

BrdU+ cells in the sub granular zone (SGZ) of the dentate gyrus (DG) were counted manually on a Leica CTR5500 microscope (40× objective) using the Leica MM AF program (MetaMorph version 1.6.0, Nashville, TN, USA). DCX+ cells were manually counted in the SGZ and granular cell layer (GCL) of the DG on a Zeiss Axiophot light microscope (40× objective) with Microfire camera using StereoInvestigator software (MBF Bioscience, Williston, VT, USA). DCX+ cells were further classified for DCX+ cell maturation based on the morphological appearance; type I, horizontal cells without a process reflected immature, mitotic cells, type II cells with an apical process into the GCL reflected an intermediate stage and type III cells with a dendritic tree reaching to the molecular layer reflected the immature neuronal stage (Hoeijmakers et al., 2018).

### Statistics

Statistical analysis was performed using SPSS 20.0 (IBM software) and Graphpad Prism 5 (Graphpad software). Data were considered statistically significant when *p* < 0.05. All graphical representation of data shows mean + standard error of the mean (SEM).

Data was analyzed with condition (Ctrl-ES) and genotype (WT-APP/PS1) as independent factors in two-way analysis of variance (ANOVA) designs. Mixed statistical models with litter and/or cohort (applicable to MWM data and brain measurements at 10 months) included as random factor were run to assess whether litter or cohort effects influenced the dependent variables. Locomotion behavior over days in the ORT or OLT, and MWM escape latency was assessed using repeated measure ANOVAs, with “days” as the repeated factor. Exploration ratios in the ORT and OLT, and probe trial analyses were tested per experimental group using a one-sample *T-*test to assess the difference from “1” (equal ratio) or “25%” (chance level performance) respectively. Pearson’s correlation was employed for the inter-parameter correlations of brain tissue measurements and/or behavioral outcome.

## Results

### ES Does Not Accelerate the Onset of Cognitive Impairments in Young Adult APP/PS1 Mice

#### ES and APP/PS1 Overexpression Do Not Affect Cognitive Functioning at 2 Months

**Figure 1:**
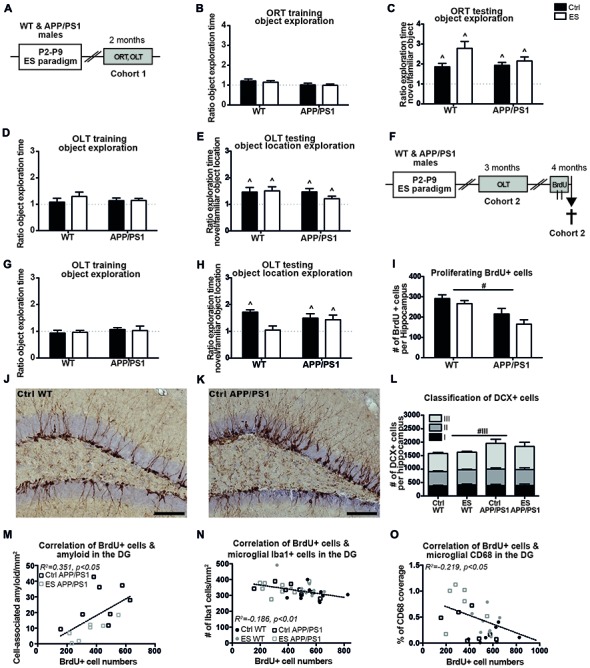
APP/PS1 mice fail to show an early-life stress (ES)-induced cognitive deficit at 3 months and exhibit altered hippocampal neurogenesis at 4 months. **(A)** C57BL/6J wild type (WT) mice and APP/PS1 male littermates (cohort 1) were exposed to ES or the control (Ctrl) condition from postnatal day (P)2 to P9 and cognitive performance was assessed at 2 months, using the object recognition task (ORT) and object location task (OLT). At 2 months, **(B)** the mice equally explored both objects during training and **(C)** explored the novel object more than the familiar object. **(D)** Object location exploration during training was equal to a ratio of 1 and **(E)** all groups explored the novel location more. **(F)** WT and APP/PS1 mice of cohort 2 were exposed to ES and tested in the OLT at 3 months of age. Mice were injected with 6 injections of 100 mg/kg 5-bromo-20-deoxyuridine (BrdU) over 2 days and sacrificed 2 h after the last injection for the analysis of BrdU+ and Doublecortin (DCX) + cells. **(G)** At 3 months, all mice explored both objects equally during OLT training. **(H)** ES WT mice were not able to discriminate between the novel and familiar object location, while other groups explored the novel object location more. **(I)** Proliferating BrdU+ cells in the hippocampus were reduced in APP/PS1 mice. DCX+ cells in **(J)** a Ctrl WT mouse and **(K)** a Ctrl APP/PS1 mouse. **(L)** DCX+ type 3 immature neurons were increased in APP/PS1 mice. BrdU+ cell numbers correlated with **(M)** the previously reported cell-associated amyloid levels, **(N)** microglial Iba1 + cells and **(O)** microglial CD68 coverage in the dentate gyrus (DG). Scale-bars are 100 μm. Annotations: ^∧^sig from 1; ^#^genotype effect. Abbreviations: Ctrl, control; DCX, doublecortin; DG, dentate gyrus; ES, early-life stress; ORT, object recognition task; OLT, object location task; P, postnatal day; SGZ, sub granular zone; WT, wild type.

Cognitive functioning was first addressed in 2-month-old mice (belonging to cohort 1) to test for an accelerated onset of cognitive impairments (Figure 1A). The mice showed equal exploration of the objects during the training phases of the ORT at 2 months of age (*ORT object exploration ratio Ctrl WT t_(6)_ = 2.114, p = 0.079, ES WT t_(6)_ = 1.875, p = 0.110, Ctrl APP/PS1 t_(12)_ = 0.135, p = 0.895, ES APP/PS1 t_(14)_ = 0.1557, p = 0.879*; Figure 1B). All mice were able to discriminate between the novel and familiar object in the ORT (*Ctrl WT t*_(6)_ = *3.951, p < 0.001, ES WT t*_(6)_ = *4.981, p = 0.003, Ctrl APP/PS1 t*_(12)_ = *6.180, p < 0.001, ES APP/PS1 t*_(14)_ = *5.789, p < 0.001*; Figure 1C). During the OLT training phase, the mice explored both objects equally (*Ctrl WT t*_(8)_ = *0.551, p = 0.597, ES WT t*_(8)_ = *1.769, p = 0.115, Ctrl APP/PS1 t*_(12)_ = *1.378, p = 0.193, ES APP/PS1 t*_(14)_ = *1.816, p = 0.09*; Figure 1D) while on the testing day, Ctrl and ES mice of both genotypes explored the novel location of the object more than the familiar location (*Ctrl WT t*_(8)_ = *2.697, p = 0.027, ES WT t*_(8)_ = *3.304, p = 0.011, Ctrl APP/PS1 t*_(12)_ = *3.811, p = 0.003, ES APP/PS1 t*_(14)_ = *2.243, p = 0.042*; Figure 1E).

#### Spatial Memory Is Impaired by ES at 3 Months of Age in WT Mice, But Not in APP/PS1 Mice With or Without ES Exposure

A second cohort (cohort 2) was tested for cognitive performance in the OLT at the age of 3 months (Figure 1F). Three-month-old mice of both conditions and genotypes equally explored the two objects during the OLT training phase (*Ctrl WT t_(9)_ = 0.650, p = 0.532, ES WT t_(10)_ = 0.572, p = 0.580, Ctrl APP/PS1 t_(8)_ = 0.972, p = 0.360, ES APP/PS1 t_(7)_ = 0.160, p = 0.876*; Figure 1G). ES exposure impaired discrimination of the novel location in WT mice only (*ES WT t*_(10)_ = *0.311, p = 0.762*), while all other experimental groups (Ctrl WT mice and Ctrl and ES APP/PS1 mice) explored the novel object location more than the familiar object location during the testing day (*Ctrl WT t*_(9)_ = *8.421, p < 0.001, Ctrl APP/PS1 t*_(8)_ = *2.954, p = 0.018, ES APP/PS1 t*_(7)_ = *2.517, p = 0.040*; Figure 1H).

#### Markers of AHN Are Altered in APP/PS1 Mice, But Not ES Mice, at 4 Months of Age

One month after the mice of cohort 2 underwent behavioral testing in the OLT, the process of AHN was assessed at the age of 4 months. BrdU+ proliferating cells in the hippocampal SGZ were found to be reduced in APP/PS1 mice, without any interaction between condition and genotype (*genotype F*_(1,33)_ = *19.450, p < 0.001, condition F*_(1,33)_ = *3.471, p = 0.071, interaction F*_(1,33)_ = *0.413, p = 0.525*; Figure 1I). Representative images show the DCX+ cells in Ctrl WT (Figure 1J) and Ctrl APP/PS1 (Figure 1K) mice. The total number of DCX+ cells in the whole granular zone (i.e., the SGZ and GCL) was not significantly affected by either the early-life condition or genotype (*genotype F*_(1,32)_ = *2.962, p = 0.095, condition F*_(1,32)_ = *0.043, p = 0.840, interaction F*_(1,32)_ = *0.219, p = 0.643*; Figure 1L).

Further classification of the different stages of DCX+ cell maturation, as based on their morphological appearance, indicated that type III DCX+ immature neurons were increased in APP/PS1 mice compared to WT mice, without an effect of ES in either of the genotypes (*type I: genotype F_(1,32)_ < 0.001, p = 0.985, condition F_(1,32)_ = 0.352, p = 0.557, interaction F_(1,32)_ = 0.293, p = 0.592; type II: genotype F_(1,32)_ = 1.455, p = 0.237, condition F_(1,32)_ = 0.006, p = 0.941, interaction F_(1,32)_ = 0.292, p = 0.593; type III: genotype F_(1,32)_ = 5.897, p = 0.021, condition F_(1,32)_ = 0.369, p = 0.548, interaction F_(1,32)_ = 0.134, p = 0.717*; Figure 1L).

In addition, the changes in BrdU+ and DCX+ cell numbers did not correlate with the behavioral performance in the OLT at 3 months of age (*OLT vs*. *BrdU: r = 0.009, p = 0957; OLT vs. DCX: r = 0.051, p = 0.763)*.

#### Aβ and Microglial Markers Correlate With Markers for the Process of AHN at 4 Months

We had previously analyzed and reported on how ES affected β-amyloid pathology, Iba1 and CD68 in the hippocampus in APP/PS1 mice (Hoeijmakers et al., 2017), using the same 4-month-old mice as used in the current study. We therefore tested to what extent the cell numbers and OLT behavior correlate with these measures. In 4-month-old mice, the number of BrdU+ cells correlated positively with the extent of cell-associated amyloid in the DG of APP/PS1 mice (*BrdU vs. cell-associated amyloid: r = 0.593, p = 0.015*; Figure 1M). In addition, BrdU+ cell numbers in 4-month-old mice correlated with microglial Iba1 + cell numbers in the DG and CD68 coverage in the DG (*BrdU vs. Iba1: r = −0.432, p = 0.008*; Figure 1N*; BrdU vs. CD68: r = 0.468, p = 0.012*; Figure 1O). Interestingly, the analysis of solely the WT mice also revealed a negative correlation of BrdU with Iba1 (*WT BrdU vs. Iba1: r = −0.490, p = 0.024*).

### APP/PS1 and ES-Exposed Mice Showed Hyperactive Exploration in ORT, OLT and EPM at 8 Months of Age

#### ES WT and APP/PS1 Groups Exhibit Abnormal Exploratory Behavior During Habituation and Testing of the ORT and OLT

**Figure 2:**
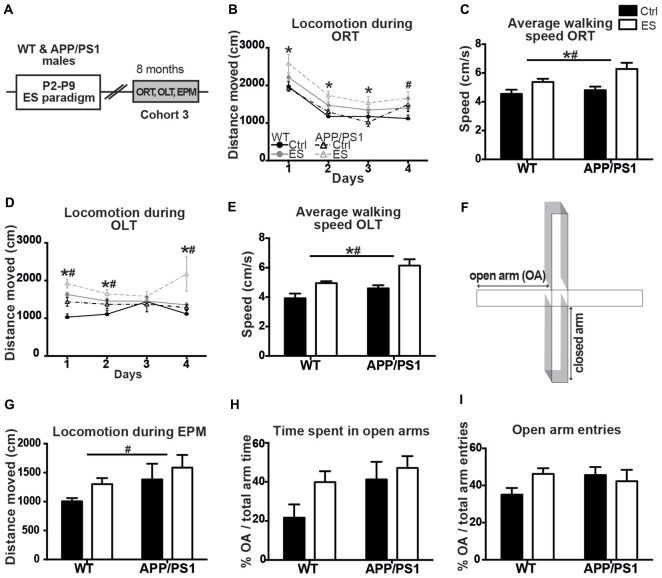
ES and APP/PS1 lead to hyperactive exploratory behavior in multiple tasks. **(A)** WT and APP/PS1 mice (cohort 3) were exposed to ES and studied in the ORT, OLT and elevated plus maze (EPM) at 8 months. **(B)** Distanced moved during box exploration for the ORT was higher for ES mice on days 1–3, and higher for APP/PS1 mice on day 4. **(C)** Average walking speed of both the ES and APP/PS1 mice was increased during these exploration days. **(D)** Locomotion was similarly higher for ES and APP/PS1 mice on days 1, 2 and 4 of OLT box exploration and **(E)** average speed was likewise increased in ES and APP/PS1 mice. **(F)** Schematic of the EPM setup, with two closed arms, two open arms (OA). **(G)** Walking distance in the EPM was elevated in APP/PS1 mice. **(H)** OA time or **(I)** number of OA entries was not different between the 4 groups. Annotations: *condition effect; ^#^genotype effect. Abbreviations: Ctrl, control; ES, early-life stress; EPM, elevated plus maze; OA, open arm; ORT, object recognition task; OLT, object location task; P, postnatal day; SGZ, sub granular zone; WT, wild type.

A third cohort of mice of all four experimental groups (cohort 3) was aged until 8 months with the aim to test if ES would have aggravated cognitive deficits in APP/PS1 mice (Figure 2A). However, all ES and APP/PS1 mice showed abnormal exploration behavior during ORT and OLT habituation and testing phases, when compared to Ctrl WT mice at this age, as indicated by the abnormal mobility patterns. This precluded any conclusions about cognitive functioning based on these tasks.

Ctrl WT mice reduce their mobility in the ORT-box over the 4 days of sequential habituation, training and testing, but locomotion was overall higher in ES WT mice and similarly elevated in APP/PS1 mice on the last day (*days F_(1,17)_ = 82.758, p < 0.001, genotype F_(1,17)_ = 3.271, p = 0.088, condition F_(1,17)_ = 13.251, p = 0.002, interaction F_(1,17)_ = 0.064, p = 0.317; *post hoc*: condition Day (D)*1 *p = 0.006, condition D*2 *p = 0.003, condition D*3 *p = 0.010, genotype D*4 *p = 0.016*; Figure 2B). In line with this observation, the average moving-speed during the ORT was increased in both ES-exposed groups (*genotype F*_(1,17)_ = *3.288, p = 0.088, condition F*_(1,17)_ = *13.108, p = 0.002, interaction F*_(1,17)_ = *1.032, p = 0.317*; Figure 2C).

Similarly, general exploration behavior was unequal between groups in the OLT during the 4 days, with both ES-exposed and APP/PS1 mice showing elevated locomotion activity (*days F_(1,17)_ = 0.849, p = 0.473, genotype F_(1,17)_ = 10.906, p = 0.004, condition F_(1,17)_ = 15.060, *p = 0.001, interaction F*_(1,17)_ = 0.515, p = 0.483; *post hoc*: genotype D*1 *p = 0.007, condition D*1 *p < 0.001, genotype D*2 *p = 0.051, condition D*2 *p = 0.018, genotype D*4 *p = 0.020, condition D*4 *p = 0.023*; Figure 2D). Next to this, the average walking speed was elevated in both ES and APP/PS1 groups (*genotype F*_(1,17)_ = *11.402, p = 0.004, condition F*_(1,17)_ = *15.470, p = 0.001, interaction F*_(1,17)_ = *0.466, p = 0.504*; Figure 2E).

#### Exploratory Behavior of APP/PS1 Mice in the EPM Is Atypical With Increased Open Arm Exploration, Whereas ES Mice Fail to Show Any Alteration

These findings on hyperactivity prompted us to test these same mice for anxiety-related exploration behavior in the EPM (Figure 2F). Locomotion in the EPM was higher in the APP/PS1 mice compared to the WT groups, while ES did not affect this (*OA time* *genotype F*_(1,16)_ = *5.399, p = 0.034, condition F*_(1,16)_ = *2.520, p = 0.132, interaction F*_(1,16)_ = *0.046, p = 0.832*; Figure 2G). However, the time spent in the OAs of the EPM, as well as the percentage of OA entries did not differ between groups (*genotype F*_(1,16)_ = *3.523, p = 0.078, condition F*_(1,16)_ = *2.8311, p = 0.112, interaction F*_(1,16)_ = *0.741, p = 0.402*; Figure 2H; *genotype F*_(1,16)_ = *0.567, p = 0.462, condition F*_(1,16)_ = *2.703, p = 0.120, interaction F*_(1,16)_ = *0.779, p = 0.390*; Figure 2I).

### Cognition and Markers of the AHN Process Are Impaired in 9-Month-Old APP/PS1 Mice and Not Further Modulated by Previous ES Exposure

#### MWM Acquisition and Reversal Training Is Impaired in APP/PS1 Mice and ES Did Not Modulate This in Mice of Both Genotypes

**Figure 3:**
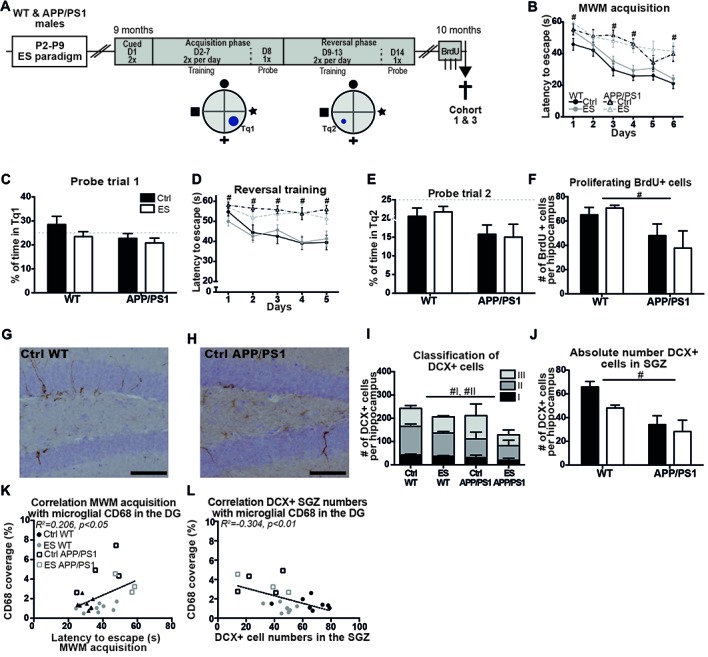
Spatial memory and adult hippocampal neurogenesis (AHN) are impaired in middle-aged APP/PS1 mice. **(A)** WT and APP/PS1 mice (cohort 1 and 3) were exposed to ES and tested in the Morris water maze (MWM) at 9 months. A schematic overview illustrates the MWM protocol that consisted of 14 days (D) with first cued trials, an acquisition phase with the platform in target quadrant (Tq) 1 and a reversal phase with platform in Tq2. One month later, BrdU was injected prior to sacrifice. **(B)** WT mice reduced their escape latency over the days of acquisition training, but APP/PS1 were slower in this. **(C)** The percentage of time spent in Tq1 was not above chance level (25%) for any of the groups during probe trial 1. **(D)** Escape latency during reversal training was slower in APP/PS1 mice compared to WT mice. **(E)** None of the groups performed above chance level during probe trial 2. **(F)** BrdU+ cell numbers were reduced in APP/PS1 mice. DCX + cells in **(G)** a Ctrl WT mouse and **(H)** a Ctrl APP/PS1 mouse. **(I)** DCX+ subtypes I and II were reduced in APP/PS1 mice. **(J)** In the sub granular zone (SGZ), the number of DCX+ cells was reduced by APP/PS1. Previously reported CD68 coverage in the DG correlated with **(K)** MWM escape latency and **(L)** DCX+ cell numbers in the SGZ. Scale-bars are 100 μm. Annotations: ^#^genotype effect. Abbreviations: Ctrl, control; D, day; DCX, Doublecortin; DG, dentate gyrus; ES, early-life stress; MWM, Morris water maze; P, postnatal day; SGZ, sub granular zone; Tq, target quadrant; WT, wild type.

Behavior of the mice from cohorts 1 and 3 was tested in the MWM at 9 months of age (Figure 3A). Overall, the swimming speed of the mice was not different between the groups during the whole MWM protocol (*genotype F_(1,51)_ = 1.602, p = 0.211, condition F_(1,51)_ = 0.945, p = 0.336, interaction F_(1,51)_ = 0.272, p = 0.605*). APP/PS1 mice were significantly slower in locating the platform than WT groups, even though all groups showed a reduced latency to escape during the course of the MWM acquisition (*days F*_(1,51)_ = *19.846, p < 0.001, genotype F*_(1,51)_ = *23.198, p < 0.001, condition F*_(1,51)_ = *2.297, p = 0.136, interaction F*_(1,51)_ = 0.154, p = 0.696; post hoc for day*genotype: Acq1 *p = 0.002, Acq*3 *p < 0.001, Acq*4 *p = 0.001, Acq*6 *p < 0.001*; Figure 3B). Although all mice acquired the task, none of the groups spent above 25% (chance level) of the time in Tq1 during the probe trial (*Ctrl WT t*_(13)_ = *1.031, p = 0.321; ES WT t_(19)_ = 0.723, p = 0.478; Ctrl APP/PS1 t*_(10)_ = *1.106, p = 0.295; ES APP/PS1 t*_(9)_ = *2.093, p = 0.065*; Figure 3C).

During the more challenging reversal training, APP/PS1 mice were slower to locate the platform during all days of reversal training than WT mice (genotype F_(1,51)_ = 29.985, p < 0.001, condition F_(1,51)_ = 0.434, p = 0.513, interaction F_(1,51)_ = 0.281, p = 0.598; *post hoc* for day*genotype: rev1 *p = 0.026, rev*2 *p = 0.003, rev*3 *p = 0.001, rev*4 *p < 0.001, rev*5 *p = 0.001*; Figure 3D). Only Ctrl WT mice showed a reduction in escape latency over the different training days (*rev days F*_(1,51)_ = *5.294, p < 0.001; Ctrl WT F*_(3.23,42.04)_ = *4.809, p = 0.005; ES WT F*_(3.10,58.90)_ = *2.326, p = 0.082; Ctrl APP/PS1 F*_(1.95,19.48)_ = *0.684, p = 0.513; ES APP/PS1 F*_(2.74,24.65)_ = *0.586, p = 0.615*; Figure 3D). During the probe trial that followed reversal training, none of groups displayed memory for the new location as all of the groups spent less than 25% of the time in the Tq2 (*Ctrl WT t*_(13)_ = *2.006, p = 0.066; ES WT t*_(19)_ = *3.689, p = 0.004; Ctrl APP/PS1 t*_(10)_ = *2.243, p = 0.037; ES APP/PS1 t*_(9)_ = *2.904, p = 0.018*; Figure 3E).

#### Markers for the Process of AHN Are Reduced in APP/PS1 Mice at 10 Months

A month after the end of the MWM paradigm, AHN was assessed in the 10-months-old mice. BrdU+ cell numbers were reduced in the SGZ of APP/PS1 mice at 10 months, but ES did not affect this in either genotype (*genotype F_(1,22)_ = 11.41, p = 0.003, condition F_(1,22)_ = 0.099, p = 0.756, interaction F_(1,22)_ = 1.168, p = 0.292*; Figure 3F). Representative images of DCX+ cells in 10-month-old WT (Figure 3G) and APP/PS1 mice (Figure 3H) and quantification showed that the total number of DCX+ cells was not affected in APP/PS1 mice at this age (*genotype F*_(1,22)_ = *1.746, p = 0.200, condition F*_(1,22)_ = *2.049, p = 0.166, interaction F*_(1,22)_ = *0.308, p = 0.585*; Figure 3I). The morphological classification of DCX+ cells showed that the type I proliferative and type II intermediate maturation stages were reduced in APP/PS1 compared to WT mice, while ES did not further influence this reduction (*Type I: genotype F*_(1,22)_ = *5.853, p = 0.024, condition F*_(1,22)_ = *2.107, p = 0.161, interaction F*_(1,22)_ = *0.247, p = 0.624; Type II: genotype F*_(1,22)_ = *6.171, p = 0.021, condition F*_(1,22)_ = *1.671, p = 0.210, interaction F*_(1,22)_ = *0.025, p = 0.876; Type III: genotype F*_(1,22)_ = *0.001, p = 0.976, condition F*_(1,22)_ = *1.803, p = 0.193, interaction F*_(1,22)_ = *1.016, p = 0.324*; Figure 3I). Similarly, region-specific analysis showed that DCX+ cells that reside in the SGZ were reduced in the APP/PS1 groups (*genotype F*_(1,22)_ = *29.402, p < 0.001, condition F*_(1,22)_ = *3.042, p = 0.095, interaction F*_(1,22)_ = *2.688, p = 0.115*; Figure 3J). Furthermore, both BrdU+ cell numbers and DCX+ cell numbers did not correlate with the behavioral performance in the MWM at 9 months of age (*MWM acq vs. BrdU: r = −0.288, p = 0.162; MWM rev vs. BrdU: r = −0.213, p = 0.307; MWM acq vs. DCX: r = −0.349, p = 0.088; MWM rev vs. DCX: r = 0.035, p = 0.867*).

#### Microglial CD68 Correlates With Cognition, BrdU and DCX SGZ Cells at 10 Months

Amyloid pathology and neuroinflammation parameters have previously been analyzed and reported for this cohort of 10-month-old mice (Hoeijmakers et al., 2017), and we now further analyzed inter-parameter correlations between these factors, and the new cognition and cell number data. At 10 months of age, BrdU+ and DCX+ cell numbers did not correlate with amyloid pathology (*BrdU vs. cell associated amyloid: r = −0.159, p = 0.682; BrdU vs. plaque load: r = −0.021, p = 0.958; DCX vs. cell associated amyloid: r = −0.144, p = 0.711; DCX vs. plaque load: r = −0.054, p = 0.890*), while acquisition training in the MWM correlated with microglial CD68 coverage in the DG of 10-month-old mice (*MWM acquisition vs. CD68: r = 0.454, p = 0.030*; Figure 3K). Microglial CD68 coverage in the DG further showed a negative correlation of DCX+ cells numbers in the SGZ, but not with total DCX+ cell numbers (*DCX+ SGZ vs. CD68: r = −0.552, p = 0.006*; Figure 3L*; Total DCX+ vs. CD68: r = −0.208, p = 0.341)*.

## Discussion

In this study, we addressed if chronic ES exposure accelerated or aggravated AD-related cognitive decline and alterations in the process of adult neurogenesis in APP/PS1 mice, a classic model for aspects of AD. Cognitive deficits have previously been indicated in ES WT mice at 4 months of age, and we now showed that these deficits were not yet present when ES-exposed mice were studied at 2 months. However, OLT performance was impaired in 3-month-old ES WT, but not ES APP/PS1 mice. At 4 months of age, the APP/PS1 mice further exhibited an increased number of differentiating DCX expressing cells, but a decrease in BrdU+ cell numbers in the SGZ. These indicators of the neurogenic process did not correlate with OLT performance of these mice at 3 months, whereas BrdU+ cell numbers were associated with the APP/PS1-induced neuropathological and microglial changes at 4 months, that we reported before Hoeijmakers et al. (2017). At 9 months of age, we confirm the learning impairments of APP/PS1 mice in the MWM and show that ES did not further modulate cognition of the APP/PS1 mice, nor of WT mice. The process of AHN was reduced in APP/PS1 mice at 10 months of age. Finally, DCX+ cell numbers and cognition in the 10-month-old mice correlated with the previously reported microglial CD68 coverage in the DG (Hoeijmakers et al., 2017). Together, these results show that ES does not accelerate or aggravate the AD-related cognitive decline or the alterations in AHN in APP/PS1 mice.

### No Accelerated Cognitive Decline and Neurogenic Alterations in Young Adult (2–4 Months) APP/PS1 Mice Exposed to ES

We show for the first time that ES mice were able to discriminate between novel and familiar objects at 2 months of age and that spatial memory impairments in ES WT mice had developed by 3 months. This observation is in line with, and extends on the previous descriptions of impairments in cognition in this ES model at ages 4–6 months (Rice et al., 2008; Wang et al., 2011, 2013; Naninck et al., 2015). Our results further indicate that the ES-induced cognitive deficits in novelty-based learning tasks developed only after 2 months of age. Interestingly, exposure of rats to limited nesting and bedding material from P2 to P9 similarly induced deficits at 10, but not yet at 4 months of age (Brunson et al., 2005). Although these mouse and rat models are not identical, this does imply that chronic ES induced impairments in specific cognitive tasks arise not until later in adulthood.

Although ES WT mice were impaired in the OLT at 3 months of age, ES-exposed APP/PS1 mice were still able to learn the task at this time. It is thus evident that ES does not accelerate impairments in the APP/PS1 mice at this age, as these ES-exposed transgenic mice did not show impairments yet. We suggest that this difference in the phenotype after ES exposure in WT and APP/PS1 mice is potentially mediated by alterations in the neurogenic processes. Although 4-month-old APP/PS1 mice exhibited reduced numbers of proliferating, BrdU+ cells, the number of differentiating, DCX+ cells was enhanced in APP/PS1 mice, irrespective of ES. An increase in among others DCX+ cell numbers has also been reported in 3-month-old APP/PS1 mice, as well as in other AD-related transgenic lines, in particular during the earlier pathological stages when no or little plaque formation has developed yet (Wen et al., 2002; Krezymon et al., 2013; Unger et al., 2016). This phenotype has been proposed to be a potential compensatory mechanism of the brain to combat the degenerative effects of soluble Aβ peptides (Meneghini et al., 2013). However, both the BrdU+ and DCX+ cell numbers at 4 months of age did not correlate with OLT performance at 3 months, and an open question thus remains to what extent alterations in the process of AHN contribute to cognition in the ES-exposed and APP/PS1 mice.

We further addressed whether cognitive performance and markers of the AHN process were associated with previously described changes in hippocampal Aβ levels and neuroinflammation in these mice (Hoeijmakers et al., 2017). Interestingly, BrdU+, but not DCX+, cell numbers correlated positively with cell-associated amyloid levels, and negatively with the microglial markers Iba1 and CD68 in the DG. Clearly, genotype is a strong contributor in these correlation, however, the correlation of microglial Iba1 with BrdU+ cells also remained significant in solely WT mice. This suggests that alterations in the AHN process might also be associated with ES-mediated alterations in neuroinflammatory signaling (Hoeijmakers et al., 2017). Indeed, hippocampal pro-inflammatory signaling *per se* can impact neuroplasticity, including AHN (Ekdahl et al., 2003; Jakubs et al., 2008; Maggio et al., 2013). However, whether these BrdU+ cell numbers relate to neurogenic and/or glial/astrogenic changes in the hippocampus awaits further investigation.

### Cognitive Decline in Middle-Aged, 9-Month-Old APP/PS1 Mice Is Not Aggravated by ES

The first cognitive deficits have been reported to arise in APP/PS1 mice after approximately 6 months of age (Jankowsky et al., 2005; Zhang et al., 2012; Edwards et al., 2014; Guo et al., 2015) and we therefore addressed whether ES would have aggravated cognitive performance at 9 months, when the deficits should have been firmly established. In comparison to WT mice, the APP/PS1 mice had indeed a slower learning curve during the acquisition and reversal phases. However, none of the groups fully acquired spatial memory for the platform location, as indicated by the poor probe trial performance of all four groups.

In order to better address more subtle differences between the groups, we used a smaller platform during reversal training that makes learning of the novel location more challenging and requires more precision (Vorhees and Williams, 2006). Now, APP/PS1 mice could indeed no longer locate this novel platform location, whereas Ctrl WT mice showed learning during this reversal phase. However, this strong APP/PS1-induced impairment in reversal training did not allow any possible further impairment in ES-exposed APP/PS1 mice. It is overall evident that APP/PS1 mice were significantly impaired in MWM performance at 9 months and ES did not significantly alter, or worsen this performance any further.

It is interesting that the ES-exposed WT mice were not impaired in MWM acquisition relative to Ctrl WT mice either, as was reported for MWM learning in Ctrl and ES WT mice at 5 months of age (Naninck et al., 2015). A natural decline in the cognitive abilities of Ctrl WT mice might possibly have leveled out the (earlier) difference between Ctrl and ES mice (Lindner, 1997; Koh et al., 2014), resulting in a similar learning capacity around middle-age. Indeed, Ctrl WT mice were not able to locate the platform during the probe trial either.

Although chronic ES exposure in WT mice was reported to impair ORT learning at 8 months (Rice et al., 2008), the 8-month-old ES-exposed and APP/PS1 mice in this study showed hyperactive exploratory behavior in both the ORT and OLT, that was further confirmed by the atypical (distal) OA exploration in the EPM. While others reported normal or reduced exploratory behavior of ES-exposed WT and APP/PS1 mice (Rice et al., 2008; Huang et al., 2016; Olesen et al., 2016), others have described similar (hyperactive) exploratory behaviors (Filali et al., 2011; Rodgers et al., 2012) that, however, hampered the proper assessment and evaluation of cognition with these tasks (Vogel-Ciernia and Wood, 2014; Cohen and Stackman, 2015). To our knowledge, the exact underlying pathways that are responsible for the hyperactive phenotype in APP/PS1 mice remains to be determined, although the phenotype seems to be relate to the APP metabolism and disease progression, as discussed in a recent review (Webster et al., 2014). Next to this, we can only speculate what mediates this phenotype in ES mice; hyperactivity in ES mice could potentially be associated with abnormalities in the striatum, which is among others relevant for locomotor activity (Barbera et al., 2016; Fareri and Tottenham, 2016), however, this requires further investigation.

Prior to our current study, a related report had shown a stronger impairment in MWM performance in APP/PS1 mice that were exposed to daily, 3-h maternal separation from P2–P21 compared to unstressed APP/PS1 mice (Hui et al., 2017). Next to this, a more “positive” early-life manipulation, i.e., early-life handling, was found to attenuate some cognitive impairments in 11-month-old APP/PS1 mice (Lesuis et al., 2017), and 4-month-old 3xTgAD mice (Cañete et al., 2015). Interestingly, we reported that 1-week of chronic ES enhanced plaque load in the DG (Hoeijmakers et al., 2017), whereas the APP/PS1 mice exposed to a 3-weeks-long maternal separation paradigm exhibited elevated Aβ plaque load in the total hippocampus as well as the cortex (Hui et al., 2017). Exposure to 3-week long maternal separation thus led to a more severe Aβ phenotype as well as more severe cognitive decline in APP/PS1 mice, relative to 1-week of chronic ES exposure in our current study. Considering that the level of Aβ has been implicate in the progression of cognitive decline in APP/PS1 mice, this might provide a possible explanation for the stronger impact of maternal separation stress on cognition decline (Hui et al., 2017).

Interestingly, MWM acquisition correlated with the previously studied microglial CD68 changes in the DG but not with the DG Aβ plaque load (Hoeijmakers et al., 2017), indicating that, similar to the phenotype at 4 months, the APP/PS1-induced neuroinflammatory changes might be associated with the emergence of the cognitive deficits (Dinel et al., 2011; Czerniawski and Guzowski, 2014; Guo et al., 2015; Fonken et al., 2016). Alternative behavioral protocols can be employed in future studies to address (subtler) ES-induced effects on cognition at different ages.

Next to cognitive deficits, APP/PS1 mice have been described to exhibit reduced AHN around the age of 9–10 months (Taniuchi et al., 2007; Verret et al., 2007; Niidome et al., 2008; Demars et al., 2010; Hu et al., 2010; Hamilton and Holscher, 2012), although there are expectations as well (Taniuchi et al., 2007; Hamilton and Holscher, 2012; Unger et al., 2016). The reduction in basal BrdU+ cell proliferation and DCX+ cell numbers in Ctrl and ES APP/PS1 mice at 9 months is therefore consistent with these previous studies. Furthermore, the reduction in DCX+ cell numbers correlated with the APP/PS1-induced elevation in microglial CD68 coverage in the DG (Hoeijmakers et al., 2017), whereas AHN did not correlated with MWM performance. Such a correlation suggests that the neuroinflammatory activation that follows APP/PS1 overexpression, and thus likely Aβ pathology, might be associated with a reduction of immature neurons as reflected by the changes in DCX+ cell numbers. Anti-inflammatory treatment has indeed been shown to induce pro-neurogenic effects on newborn cell survival in APP/PS1 mice (Biscaro et al., 2012), supporting such a relation between neuroinflammation and AHN.

In addition, exposure to chronic ES did not further aggravate the APP/PS1-induced reductions in AHN. Interestingly, at 6 months, neurogenic cell proliferation and differentiation in the hippocampus were not affected by ES in WT mice either (Naninck et al., 2015). We now extended on these findings at other ages, and further show that even under a pathological condition of APP/PS1 overexpression, these neurogenic stages are not affected by ES exposure. Neurogenic cell survival, an additional measurements for AHN, were decreased in 6-month-old ES WT males (Naninck et al., 2015), while APP/PS1 mice have been reported to exhibit reduced cell survival levels as well (Verret et al., 2007). This process might therefore be more susceptible to ES exposure in APP/PS1 mice and remains to be studied in future experiments. Next to this, other markers for neuroplasticity in ES-exposed APP/PS1 could be addressed to elucidate if (chronic) ES-induced changes in WT mice are indeed differently manifested in the APP/PS1 model (Aisa et al., 2009; Wang et al., 2011; Sierksma et al., 2012).

### Limitations and Future Goals

Although we did verify the presence of ES-induced cognitive impairment in young adulthood, we observed no changes after ES in the cognitive measures and parameters of the AHN process in the middle-aged (8–10 months) WT and APP/PS1 mice. As for the behavior, however, it remains to be determined if these results are due to the difficulty of the tasks, and that middle age animals might hence need easier behavioral tasks, in which control WT animals perform better, which would better allow the detection of a possible impairment by ES exposure. As for the brain plasticity aspect, levels of AHN are notably low at this age and it is thus possible that we are at a limit of detection of possible differences between groups. In future studies, addition measures of plasticity might be helpful to elucidate this further.

In addition to this, we now primarily focused on the hippocampus for the study of ES effects, and measurements of AHN in particular. Other hippocampal-based behaviors seem to be more strongly associated with neurogenesis, such as pattern separation (Deng et al., 2010) and might therefore be an additional interesting target for the study of ES effects related to cognition and AHN. In addition, other brain regions than the hippocampus are involved in the studied behaviors too. For instance, impairments in MWM reversal learning in APP/PS1 mice is thought to be primarily mediated by pathology-related prefrontal cortex impairments (Vorhees and Williams, 2006). Notably, we showed before that ES leads to region-specific differences, with e.g., significant effects on pathology and microglia in the hippocampus, but not the entorhinal cortex (Hoeijmakers et al., 2017). It will be interesting to address in the future whether other brain regions, besides the hippocampus, contribute to the ES cognitive phenotypes in APP/PS1 mice.

## Conclusion

To summarize, ES failed to accelerate or aggravate the decline in cognition and AHN in APP/PS1 mice. The severe cognitive deficits and already low neurogenic numbers in the middle-aged APP/PS1 mice might have prevented a further reduction by ES at this late age. Given the altered neuropathological Aβ levels and neuroinflammation in ES-exposed APP/PS1 mice, this raises the question whether ES exposure affects other forms of hippocampal neuroplasticity in APP/PS1 mice and aggravates the decline at other ages. Such knowledge can help to identify vulnerability for later-life cognitive decline in populations exposed to stress early in their lives, and may help to develop specified therapeutic interventions.

## Author Contributions

LH and AK designed the experiments. LH, AA, FV and JK performed the experimental work and data analysis. LH, PJL and AK wrote the article.

## Conflict of Interest Statement

The authors declare that the research was conducted in the absence of any commercial or financial relationships that could be construed as a potential conflict of interest.
